# Global overview of government-endorsed nutrition labeling policies of packaged foods: a document review

**DOI:** 10.3389/fpubh.2024.1426639

**Published:** 2024-11-08

**Authors:** Ummay Afroza, Ahmad Khairul Abrar, Abira Nowar, Sheikh Mohammad Mahbubus Sobhan, Nicole Ide, Sohel Reza Choudhury

**Affiliations:** ^1^Department of Epidemiology and Research, National Heart Foundation Hospital and Research Institute, Dhaka, Bangladesh; ^2^Resolve To Save Lives (RTSL), New York, NY, United States

**Keywords:** nutrition labeling, packaged food, front-of-pack labeling, labeling policy, food labeling

## Abstract

**Introduction:**

Nutrition labeling provides nutritional information about nutrients present in a food product. It is commonly applied to packaged foods and beverages, where the information can be presented on the back or front of the pack as the nutrient declaration, nutrition and health claims, and supplementary nutrition information. Nutrition labeling is an important policy instrument for improving the nutritional quality of foods and promoting healthy diets, as it allows consumers to make informed purchasing decisions. This document review aims to provide a comprehensive overview of government-endorsed nutrition labeling policies related to nutrient declaration, nutrition claims, and supplementary nutrition information enforced worldwide.

**Methods:**

We searched two nutrition policy databases, the Global database on the Implementation of Food and Nutrition Action (GIFNA) and the NOURISHING database, and government websites of some selected countries for the government-endorsed nutrition labeling policies published up to June 2023. We narrated the policy adopting countries' distribution by WHO regions, mode of implementation (voluntary or mandatory), and types of front-of-pack labels implemented.

**Results:**

Globally, we found that 95 countries have mandatory policies for nutrient declarations on packages of processed products. These include 41 countries in Europe, 19 in America, 14 in the Western Pacific, nine in Africa, seven in the Eastern Mediterranean region, and five countries from South-East Asia. Additionally, 71 countries have policies on the use of nutrient claims like “fat-free,” “excellent source,” and “fortified.” European region has the highest number of countries (37) that have rules on nutrient claims. Front-of-pack labeling (FOPL) policies have been introduced in 44 countries as supplementary nutrition information. Of these, 16 countries have adopted FOPL as mandatory, while others have implemented it voluntarily. The FOPL systems include warning labels, keyhole logo, health star rating, traffic light labeling, nutri-score, and healthy choice logos.

**Conclusion:**

Over recent years, the number of countries adopting mandatory nutrition labeling policies, especially FOPLs, has increased globally. Labeling policies should be evidence-based and follow the best practices to protect consumers from unhealthy nutrients and promote healthy eating. FOPL designs need to be selected based on country-specific evidence of effectiveness and appropriateness, avoiding industry influence.

## 1 Introduction

Unhealthy diets are one of the leading causes of morbidity and disability globally (1). Rapid urbanization, expansion of processed products industries, and lifestyle changes have resulted in transitions in habitual dietary patterns worldwide. People are now shifting from their homemade traditional diets toward a diet high in processed food products, which are calorie-dense, high in carbohydrates, saturated fat, trans fat, salt, and added sugars (2). Such dietary practices are major contributors to obesity, cardiovascular disease (CVDs), high blood pressure, type 2 diabetes mellitus, cancers, and other diet-related non-communicable diseases (NCDs) (3, 4). Every year, around 17.9 million people die from CVDs, which comprises 32% of the total worldwide deaths (5). According to the Global Burden of Disease (GBD) 2019 report, dietary risk factors resulted globally in around 8 million deaths and 188 million disability-adjusted life years (DALYs) from CVDs each year (6). This dietary risk encompasses the inadequate intake of healthy-nutrient-rich foods like fruits, vegetables, legumes, nuts, and seafood with simultaneous excessive intake of calorie-dense processed products and foods having nutrients that are harmful to health such as added sugar, sodium, and trans-fatty acid (7).

Nutrition labeling is an important policy instrument for reducing malnutrition in all its forms, improving the nutritional quality of foods, and promoting healthy diets (8). Nutrition labeling has been recommended in several global agreements, approved by the World Health Assembly (WHA) since it provides the nutritional content of foods allowing consumers to make informed purchasing decisions (9, 10). Nutrition labeling may include ingredient lists, nutrient declarations, supplementary nutrient information, and nutrition and/or health claims. The nutrient declaration, more commonly known as Nutrition Fact Panel (NFP) and typically placed in the Back-of-Pack (BOP), is a standardized statement that lists nutrients (i.e., energy value, protein, available carbohydrate, fat, saturated fat, sodium, total sugar) present in the food along with their quantities. Supplementary nutrient information, like front-of-package labeling (FOPL), is a way to enhance consumers' understanding of the nutritional quality of food and to facilitate their interpretation of the nutrient declaration (11, 12). A nutrient claim means any representation that provides information about certain nutritional properties of a food product (e.g., “low sodium”). Health claims imply a relationship between a food or the consistency of that food and health (e.g., “heart-healthy”) (13). As many countries do not regulate these claims, companies often use nutrition and health claims without scientific basis, thus potentially creating a “health halo” to lead consumers into thinking their product is healthy, which may not be the case (14).

As part of multiple strategies for marketing restrictions on unhealthy processed products, the World Health Organization (WHO) has urged its member states to implement a government-led mandatory FOPL system as one of the “best buys” to promote healthy diet and control and prevent the burden of NCDs (15, 16). Food companies deliberately add misleading nutrition claims (e.g., “low fat” or “high in vitamins”) to create a “health halo effect,” which leads consumers to perceive unhealthy food products as healthier than they truly are. This can obscure the products' actual nutritional quality (17, 18). In such cases, FOPL provides consumers with nutrition information in a clearer and more understandable format. A growing body of evidence suggests that FOPLs can facilitate consumers' understanding of food nutritional quality, supporting healthier food choices. These labels guide consumers to compare similar products easily and help consumers avoid less nutritious processed products. By providing nutritional information, FOPLs empower consumers and encourage the selection of healthier options. Furthermore, it can also prompt processed products reformulation by respective industries to improve quality and appeal to health-conscious consumers and avoid negative marketing implications, such as by reducing trans-fat and sodium content (19–21). Multiple FOPL systems have been introduced by different countries, including warning labels, summary labels, or a combination of both logos and factual declarations (22, 23). FOPLs can be grouped into different types: informative/non-interpretative and interpretative (informative/non-interpretative FOPL provides factual information, whereas interpretative FOPL provides evaluative judgment on nutritional quality); directive, non-directive, and semi-directive labels (based on the degree to which labels provide direct judgment about healthiness); nutrient-specific systems (focusing on specific nutrient concentrations) and nutrient summary systems (providing an overall healthfulness score) (11).

The purpose of this document review is to offer a comprehensive overview of available government-endorsed labeling policies around the world. The review aims to synthesize existing nutrition labeling policies, including nutrient declarations, rules on nutrient claims, and FOPL schemes.

## 2 Methods

To conduct this document review, we searched two policy databases, the Global Database on the Implementation of Food and Nutrition Action (GIFNA) (23) of the World Health Organization (WHO) and NOURISHING policy databases of the World Cancer Research Fund International (22, 23). The search was conducted during the period of July to October 2023 to identify the nutrition labeling policies published/enforced up to June 2023 in different countries around the world. The GIFNA database has “front-of-pack and other interpretive nutrition labeling” and “country score card” for sodium and sugar, which document countries' progress in implementing supplementary nutrition labels (FOPL and other interpretive labels) and declaration of sugar and sodium on the packaged food. The NOURISHING database contains information on policies regarding food labeling and restrictions on products high in sugar, salt, and fat. We used a list of keywords for searching the databases: the keywords were “nutrition labeling,” “front-of-pack labeling,” “warning label,” “law,” “legislation,” and “policies.” The keywords were translated into the country's respective languages for countries with policies in their native languages. In addition, we double-checked the search results by looking through the national websites and publicly available resources on Google, such as official and intergovernmental reports.

We developed and followed relevant inclusion and exclusion criteria for our research questions (Table 1) to provide an overview of government-endorsed labeling policies around the world. In this review, we included only the policies endorsed by the government and excluded the initiatives introduced by non-government private institutions, civil society organizations, or the food industry as they are often self-regulatory and are enforced with very limited uptake. Regarding the claims, we included policies on nutritional claims only, as other claims, health and risk reduction claims, are often confusing for the consumers and misleading.

**Table 1:** Inclusion and exclusion criteria of study selection.

**Inclusion criteria**	**Exclusion criteria**
1. Government-endorsed policies regarding nutrition labeling on packaged foods and/or beverages, including: - Front-of-pack labeling (FOPL) - Back-of-pack labeling (BOPL) including nutrient declaration of sugar, salt, protein, and fat - Policies to restrict nutrient claims	1. Policies on allergen labeling 2. Policies focusing on only labeling ingredient lists 3. Policies focusing only on health claims 4. Policies on menu labeling

We reviewed the policies selected according to the predefined criteria to describe the geographical distribution of the policy-adopting countries according to the WHO regions, types of implementation– voluntary or mandatory, and the types of schemes for the front-of-pack labels. The policy documents that were not in English were translated using Google Translate.

In this review, we organized the available nutrition labeling policies under three categories: (1) nutrient declaration, which included listing of ingredients and nutrients, (2) nutrition claims, which included content and comparative nutrient claims, and (3) supplementary nutrition information, which included front-of-pack nutrition labeling. To describe the types of front-of-pack nutrition labels, we classified the available FOPL schemes based on their approach to providing information about processed products into three types: (a) non-interpretative or reductive, which provide nutrient content information only with no specific judgment on the overall nutritional value of the food, (b) interpretative or directive system, which provide guidance on the relative healthfulness or unhealthfulness of the food, and (c) hybrid systems, which provide both nutrient content information with numbers and judgment on the nutritional values with additional colors or symbols (Table 2).

**Table 2:** Typology of nutrition- and health-related front-of-pack labels.

**Non-interpretative/reductive**	**Interpretative/directive**	**Hybrid**
Provides only nutritional information (e.g., calorie value, sugar, and fat) rather than information on the overall healthiness of a product	Provides summary information on overall healthiness or unhealthiness of the product depending on set criteria for different food categories. It does not give any specific nutrient information and gives	Provides specific nutritional information about product's and it's overall level of healthiness based on pre-determined parameters or algorithms set by nutrition experts
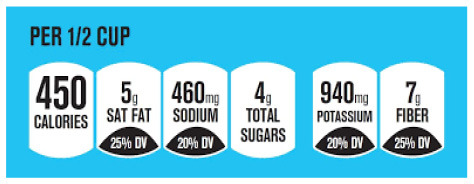 Facts up front plus nutrients to encourage	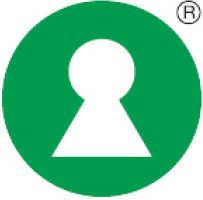 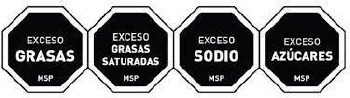 Nordic keyhole Warning labels	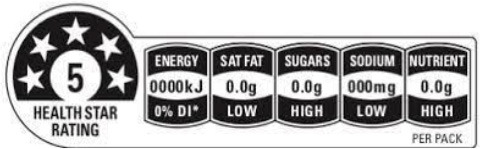 Health star rating
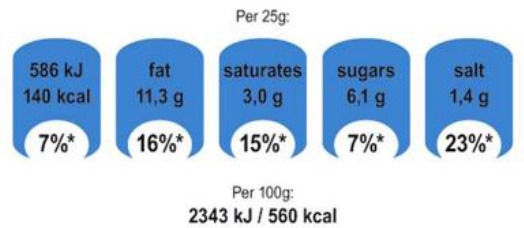 Reference intakes	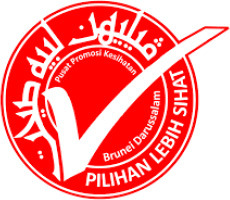 Healthy choice logo 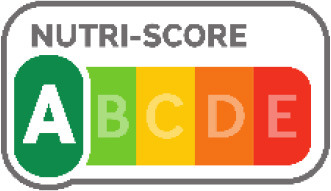 Nutri-score	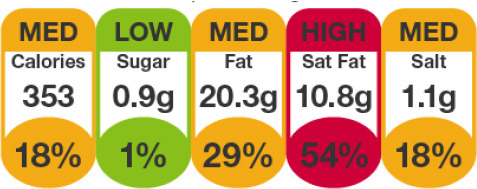 Multiple traffic light
	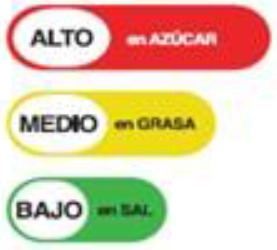 Traffic light label	

## 3 Results

This document review presents an overview of the nutrition labeling policies adopted by different countries around the world.

### 3.1 Nutrient declaration

Table 3 provides an overview of countries that have adopted mandatory policies for nutrient declaration. A total of 95 countries have policies on nutrient declaration. By region, the highest in Europe (41), followed by America (19), the Western Pacific (14) and Africa (nine). Eastern Mediterranean (seven) and South-East Asia (five) are the regions with the lowest countries having a nutrient declaration policy.

**Table 3:** Countries with the mandatory nutrient declaration worldwide (*n* = 95).

**WHO regions**	**Countries**
Africa (*n* = 9)	Algeria, Benin, Ethiopia, Seychelles, South Africa, Tanzania, Tunisia, Zambia, Zimbabwe
America (*n* = 19)	Argentina, Bahamas, Bolivia, Brazil, Canada, Chile, Colombia, Costa Rica, Ecuador, El Salvador, Guatemala, Honduras, Mexico, Nicaragua, Paraguay, Peru, Uruguay, USA, Venezuela
Europe (*n* = 41)	Austria, Belarus, Belgium, Bulgaria, Croatia, Cyprus, Czechia, Denmark, Estonia, Finland, France, Georgia, Germany, Greece, Hungary, Iceland, Ireland, Israel, Italy, Latvia, Liechtenstein, Lithuania, Luxembourg, Macedonia, Malta, Montenegro, Netherlands, Norway, Poland, Portugal, Romania, Russia, Serbia, Slovakia, Slovenia, Spain, Sweden, Switzerland, Turkey, Ukraine, United Kingdom
Eastern Mediterranean (*n* = 7)	Bahrain, Iran, Kuwait, Oman, Qatar, Saudi Arabia, United Arab Emirates
South-East Asia (*n* = 5)	Bangladesh, India, Indonesia, Sri Lanka, Thailand
Western Pacific (*n* = 14)	Australia, Brunei, China, Hong Kong, Japan, Kiribati, Malaysia, Mongolia, New Zealand, Philippines, Republic of Korea, Singapore, Taiwan, Uzbekistan

Most of the countries in European region are members of the European Union (EU). From 13th December 2016, EU Regulation 1169/2011 on the “Provision of Food Information to Consumers” made it mandatory to provide a list of the nutrients and information on the energy value, amounts of fat, saturates, carbohydrates, sugars, protein, and salt, mentioning by 100 g or 100 ml, for most of the pre-packaged foods on their back of packages (24). All 27 EU member countries have followed the mandatory nutrient declaration of packaged products.

In the American region, 19 countries, including the USA, Canada, and Mexico, have adopted these regulations. Seven countries in the Eastern Mediterranean region have a nutrient declaration policy. Of them, six countries—United Arab Emirates, Bahrain, Oman, Kuwait, Qatar, and Saudi Arabia—have a common regulation under the Gulf Cooperation Council (GCC); the remaining one is Iran. Five countries of South-East Asia region, including Bangladesh, have a policy of nutrient declaration. The Western Pacific region lists fourteen countries, including Australia, China, and Japan.

All the policies included a mandatory declaration of ingredients on the pre-packaged food labels, and most of them included additional requirements of mentioning nutrients and their amounts. The declaration of nutrients on the food package labels is compulsory in both developed and developing countries across diverse regions.

### 3.2 Nutrition claims

We have identified 71 countries having a policy on nutrition claims. Most of the countries with such policy are from the WHO European region (37) America (11), an equal number of countries (seven) from each of the Eastern Mediterranean and Western Pacific. Africa (six) and South-East Asia (three) have the lowest number of countries that have rules on nutrition claims. Table 4 provides an overview of the countries that have policies on nutritional claims.

**Table 4:** Countries with rules on nutrition claim (*n* = 71).

**WHO regions**	**Countries**
Africa (*n* = 6)	Algeria, Ethiopia, Ghana, Seychelles, South Africa, Zimbabwe
America (*n* = 11)	Argentina, Bolivia, Brazil, Costa Rica, El Salvador, Guatemala, Honduras, Mexico, Nicaragua, Peru, USA
Europe (*n* = 37)	Austria, Belarus, Belgium, Bulgaria, Croatia, Cyprus, Czechia, Denmark, Estonia, Finland, France, Germany, Greece, Hungary, Iceland, Ireland, Israel, Italy, Latvia, Liechtenstein, Lithuania, Luxembourg, Malta, Montenegro, Netherlands, Norway, Poland, Portugal, Romania, Russia, Serbia, Slovakia, Slovenia, Spain, Sweden, Switzerland, United Kingdom
Eastern Mediterranean (*n* = 7)	Bahrain, Iran, Kuwait, Oman, Qatar, Saudi Arabia, United Arab Emirates
South-East Asia (*n* = 3)	India, Indonesia, Sri Lanka
Western Pacific (*n* = 7)	Australia, Kiribati, Malaysia, New Zealand, Singapore, South Korea, Uzbekistan

The importance of regulating nutrition claims is to ensure that they accurately reflect the nutritional quality of a product. Under these regulations, nutrition claims are only allowed if the product meets specific nutrient profile criteria, such as limits on sugar, fat, or sodium content. This helps consumers make informed decisions based on trustworthy information, reducing the risk of being misled by claims that do not align with the overall healthfulness of the product.

### 3.3 Front-of-package labeling policies

In this review, we found that 44 countries have a government-endorsed policy for front-of-pack labeling (FOPL) schemes. Of them, 16 countries have adopted FOPL as mandatory, while the remaining have implemented it voluntarily (Table 5).

**Table 5:** FOPL schemes adopted and implemented worldwide (*n* = 44).

	**Mandatory (*n* = 16)**	**Voluntary (*n* = 30)**
Africa (*n* = 1)		Zambia (44)
America (*n* = 11)	Argentina (45), Bolivia (46), Brazil (47), Canada (48), Chile (49), Colombia (50), Ecuador (51), Mexico (52), Peru (53), Uruguay (54), Venezuela (55)	
Europe (*n* = 19)	Israel (56)	Austria (57), Belgium (57), Croatia (58), Denmark (59), France (60), Germany (61), Iceland (62), Ireland (63), Lithuania (64), Luxemburg (65), Macedonia (66), Norway (67), Portugal (68), Russia (69), Spain (70), Sweden (71), Switzerland (72), United Kingdom (73)
Eastern Mediterranean (*n* = 3)	Iran (74)	Saudi Arabia (75)
		United Arab Emirates (76)
South-East Asia (*n* = 3)	Sri Lanka (77)	Indonesia (79)
	Thailand^*^ (78)	Thailand^*^ (80)
Western Pacific (*n* = 7)	Singapore^*^ (81)	Australia (82), Brunei (83), Malaysia (84), New Zealand (85), Philippines (86), Singapore^*^ (87), South Korea (88)

^*^Singapore and Thailand have both voluntary and mandatory policies on FOPL.

Mandatory FOPL policies are predominantly found in the American region, with 11 countries, including Argentina, Bolivia, Brazil, Canada, Chile, Colombia, Ecuador, Mexico, Peru, Uruguay, and Venezuela. Israel from Europe, Iran from the Eastern Mediterranean, Sri Lanka and Thailand from South-East Asia, and Singapore from the Western Pacific also have mandatory FOPL policies. Notably, Singapore and Thailand have policies that are both mandatory and voluntary for different schemes of FOPL. Countries that have adopted and implemented FOPL schemes as mandatory, require food manufacturers to adopt and display on processed products by law. This regulatory approach ensures uniformity across the food industry, aiming to provide consumers with consistent information about key nutrients, such as sugar, salt, and fat.

Countries adopting the FOPL policies voluntarily are mainly from the European region, with 18 countries implementing these policies. There is no regional standard policy for the FOPL like the nutrient declaration policy adopted by the EU and GCC. In the Western Pacific region, six countries- Australia, Brunei, Malaysia, New Zealand, Philippines, Singapore, and South Korea have adopted voluntary FOPL policy. In the African region, only Zambia has implemented a voluntary FOPL policy. Saudi Arabia and United Arab Emirates from the Eastern Mediterranean, and Indonesia and Thailand from South-East Asia have adopted the FOPL policy voluntarily. Voluntary FOPL policies allow manufacturers to choose whether to use specific labels on their products. This approach provides flexibility for companies to market healthier options without legal compulsion. Voluntary policies encourage manufacturers to adopt labels that highlight positive nutritional attributes but do not enforce uniformity across all products.

### 3.4 Types of FOPL schemes used

A comprehensive document review of FOPL systems across various countries reveals a diverse landscape of approaches designed to guide consumers in making healthier food choices regarding the nutritional content of processed products. In our review, we found four types of interpretive, one non-interpretive, and two hybrid schemes of FOPL in implementation in 44 countries worldwide. An interpretive approach has been adopted in 33 countries, a non-interpretive approach in only one country (Thailand), and a mixed/hybrid approach in 10 countries (Table 6).

**Table 6:** Description of the FOPL schemes.

**FOPL scheme**	**Country**	**Description**
**Interpretative mandatory FOPLs**		
Warning label 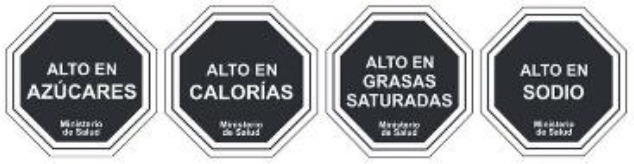	Chile (2016)	•Ministry of Health under the Food Labeling and Advertising Law (Ley 20.606) •Chile was the first country to implement the FOP warning label •Foods with high content of energy and nutrients of concern must contain an FOP black and white warning message inside a stop sign that reads “HIGH IN” •In 2019 the threshold level set for 100 g of solid was 275 kcal energy, 400 mg sodium, 10 g total sugars, and 4 g saturated fats, whereas per 100 ml liquid was 70 kcal energy, 100 mg sodium, 5 g total sugars, and 3 g saturated fats •This law also prohibits promotions and sales of products carrying warning labels to children under 14
Warning Label 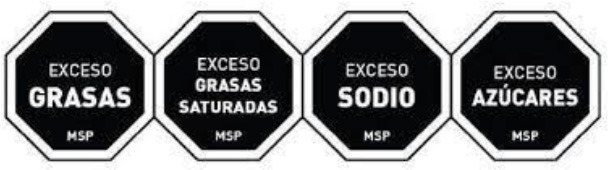	Uruguay (2018)	•Initiated by the Ministry of Health under Uruguay's Executive Decree, no. 272/18 •Implemented in March 2021 •The label requires a black and white, octagonal nutrient warning saying “HIGH IN” for products exceeding salt or sodium, fat, and sugar thresholds •When considering 100 g of food, the sodium threshold is 500 mg, sugars should make up 20% of the total calorie content, fat should account for 35%, and saturated fat for 12%
Warning Label 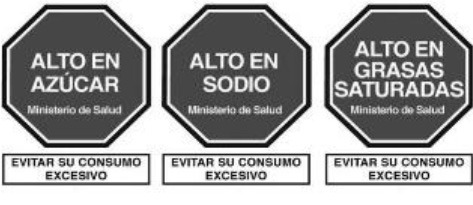	Peru (2019)	•Ministry of Health under the Law on the Promotion of Healthy Eating among Children and Adolescents •According to Supreme Decree No. 012-2018-SA, all food and beverages must contain a mandatory white or black warning label •A warning sign must be displayed if each 100 g of food includes 800 mg or more sodium, 22.5 g or more carbohydrates, or 6 g or more saturated fats. And for 100 ml of drinks 100 mg or more sodium, 6 g or more sugar, and 3 g or more saturated fats
Front-of-pack Warning Label 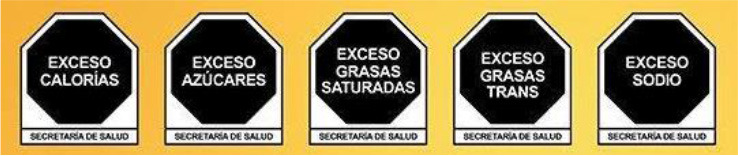	Mexico (2020)	•Mexican Health Commission (Official Standard NO. NOM-051-SCFI/SSA1-2010) •According to the government, products must contain an octagonal warning logo stating, “excessive in” if the products exceed the thresholds of sugars, saturated fats, sodium, and calories. And, rectangular warning labels if contains trans-fat •Products must contain a warning label if a food contains≥275 kcal energy for solids, ≥70 kcal total or ≥8 kcal added sugars for liquids, ≥10% total kcal from added sugars, saturated fats, ≥1 trans fats, ≥1 mg sodium per kcal or ≥300 mg, calorie-free beverage ≥45 mg sodium •If the products contain non-nutritive sweeteners or caffeine then “non recommended for children” must be displayed on labels •Child-directed marketing is prohibited for products with warning labels
Red Warning Logo 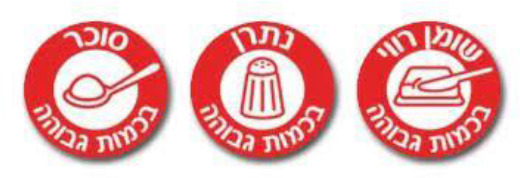	Israel (2020)	•Ministry of Health •Applicable to all industrially processed packaged solid and liquid foods •For 100 g of solid food, the limit is 400 mg of salt, 10 g of total carbohydrates, and 4 g of total saturated fat. Similarly,300 mg of salt, 5 g of total carbohydrates, and 3 g of total saturated fat is the limit for per 100 ml of beverage
Warning label 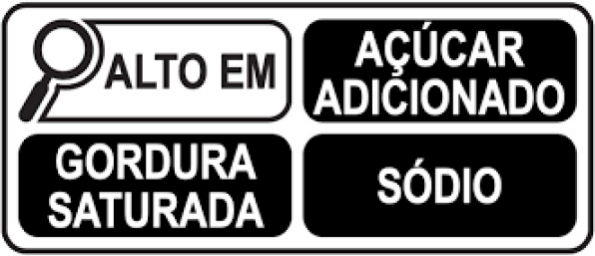	Brazil (2020)	•Brazilian Health Regulatory Agency (ANVISA) •A rectangle with a black and white magnifying glass and “high in” for added sugar, saturated fat, and sodium •The limit for 100 g of solid or semi-solid sugar is 15 g, saturated fat 6 g, and sodium 600 mg, while for 100 ml of liquid sugar is 7.5 g, saturated fat 3 g, and sodium 300 mg
Colombian Warning Label 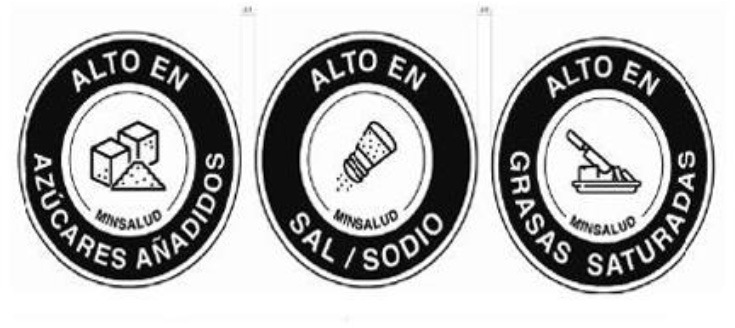	Colombia (2021)	•Ministry of Health •Circular FOPL warning labels are required for products that exceed the threshold levels •The stamp should be placed in front of the pack with a black background and white border Indicating “High in” specific nutrients. The letters of the text of the stamps must be in capital letters and white “ARIAL BOLD” font •The scheme suggests using a warning label if sodium is ≥1 mg/kcal and/or 300 mg/100 g in solid foods, and ≥1 mg/kcal in liquid items. Moreover, a warning label should also be used if the sugars and saturated fats of the food account for ≥10% of the total daily calories and ≥1% of trans fats
Warning label 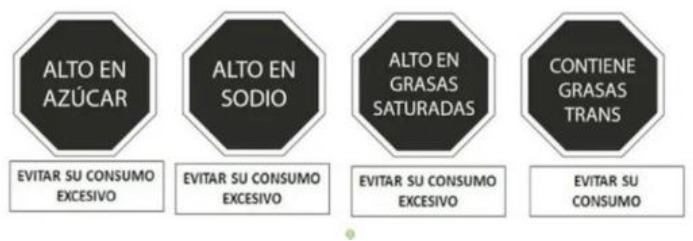	Argentina (2021)	•The National Congress of Argentina-All food and non-alcoholic beverages packaged without the consumers' presence must include warning labels if the products contain excessive amounts of salt in the final composition •The warning stamp should be a black octagon with a wide border and white capital letters and shall cover at least 5% of the package's main front •No thresholds for critical nutrients were found •Products with caffeine or non-nutritive sweeteners must include “not recommended for children” on the label
Warning Label 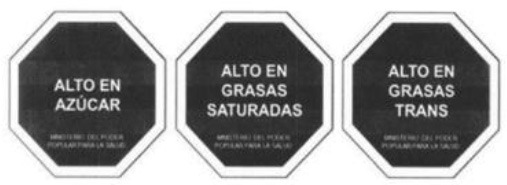	Venezuela (2021) (Not implemented yet)	•The Ministry of Health issued a resolution in December 2021, and the industry has until December 7, 2024, to implement it •The warning label must be octagonal in shape, and the text inside must say “HIGH IN” followed by “SUGAR,” “SATURATED FATS,” “TRANS FATS,” or “SALT” •Foods that have more than one label must follow the following order: (i) sugar, (ii) saturated fat, (iii) trans-fat, and (iv) sodium •The thresholds of the critical nutrients are added sugar ≥11.5 g, saturated fats ≥5 g, and ≥0 g in solid foods. For liquid foods, the thresholds are sugar ≥5.5 g, saturated fats ≥3 g, and ≥0 g
Warning label 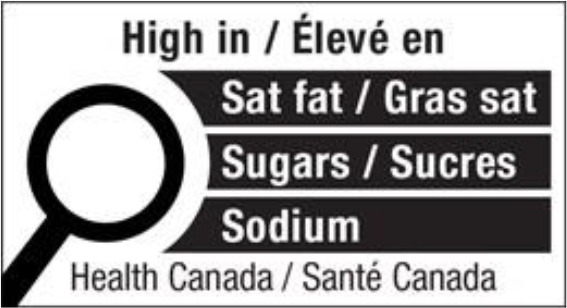	Canada (2022) (Not implemented yet)	•Health Canada introduced the nutrition labeling regulation on July 2022 •Industry has until January 1, 2026 to comply •All packaged foods that exceed saturated fat, salt, and sugar standards must have a black and white magnifying glass with the statement “high in” •No thresholds for the critical nutrients were found
Nutri-Grade Label 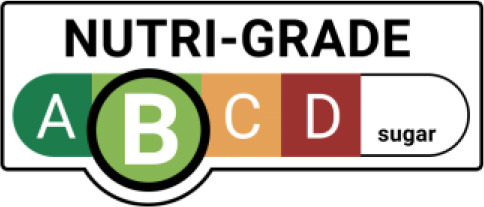	Singapore (2022)	•The Singapore government introduced Nutri-Grade labeling for beverages •Beverage will be graded using a single set of thresholds for sugar and saturated fat content with four color-coded grades (A, B, C, D). Grade C and D must contain Nutri-Grade marks on their packaging •Companies must provide nutritional information on their products •Drinks containing sugar ≤ 1 g and no sweetener per 100 ml are graded A, 2–5 g as “B,” 6–10 g as “C,” and >10 g as “D.” For saturated fats, they are classified as “A” ( ≤ 0.7 g), “B” ( ≤ 1.2 g), “C” ( ≤ 2.8 g), and “D” (>2.8 g)
Nutritional Traffic Light Label 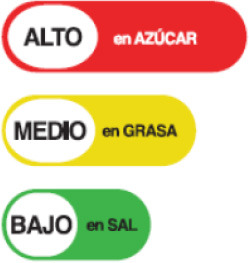	Ecuador (2014)	•Ministry of Public Health •The foods are classified into red (high), yellow (medium), or green (low) based on levels of salt, sugar, and fat •For a 100 g meal of processed food, fat ≤ 3 g, sugar ≤ 5 g, and salt ≤ 120 mg are marked green, while ≥20 g fat, ≥10–15 g sugar, and ≥600 mg salt is coded as red •In beverages, drinks containing ≤ 1.5 g fat, ≤ 2.5 g sugar, and ≤ 120 mg salt are labeled in green color whereas the drinks labeled in red contain ≥10 g fat, ≥7.5 g sugar, and ≥600 mg salt
Traffic Light Label 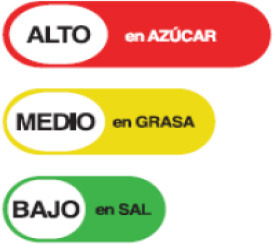	Bolivia (2016) (Not implemented yet)	•Healthy Food Promotion Law •The products are color-coded by which consumers will easily understand if it is “very high in,” “contains a moderate amount of” or is “low in” sodium or salt •The standards are advised by the Pan American Health Organization (PAHO) •No thresholds for the critical nutrients were found
**Interpretative voluntary FOPLs**		
Nordic Keyhole Logo 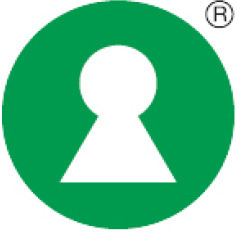	Sweden (1989), Denmark (2009), Norway (2009), Iceland (2013), Lithuania (2013), Macedonia (2015)	•Swedish National Food Agency •This logo aims to help consumers make the right choices while buying foods and stimulate the food industry to produce healthier products •The nutrient criteria for the logo are based on the “Nordic Nutrition Recommendations,” and products that can be labeled with the logo are determined by the Norwegian, Swedish, Danish, and Icelandic authorities •The logo shows that the food contains less sugar, less salt, less and healthier fats, and more dietary fibers •No thresholds for critical nutrients were found
Nutri-Score 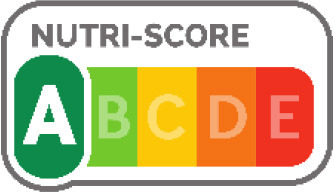	France (2017), Belgium (2018), Spain (2018), Austria (2020), Germany (2020), Luxembourg (2021), Portugal, Switzerland	•French Ministry of Health under the Health Act •The Nutri-Score method gives a final score per 100 g or 100 ml of the product •The score is calculated on the content of positive nutrients (protein, dietary fiber, calcium, and certain vitamins and minerals) and risk nutrients such as salt, sugars, saturated fat, and calories •It divides foods and beverages into five nutritional quality groups, expressed by a color spectrum ranging from dark green to dark red •No thresholds for critical nutrients were found
Good Food Logo 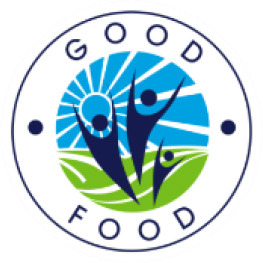	Zambia (2019)	•A joint initiative of the Government of Zambia, the SUN Business Network (SBN), the World Food Programme, and the Choices International Foundation developed the Good Food Logo in Zambia •A set of nutrition criteria based on international dietary guidelines from the World Health Organization (WHO), guides the selection of food products for consumers •The five main criteria areas are trans fats, salt, sugar, dietary fiber and micronutrients •This logo indicates that the products meet healthier nutritional standards and aims to increase consumer access to healthier options •The logo has been designed to reduce micronutrient deficiencies and prevent obesity and associated non-communicable diseases •However, no thresholds for critical nutrients have been established
Healthier Choice Logo Brunei 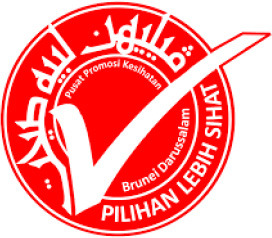	Brunei (2017)	•Ministry of Health •This logo is used for 13 food categories along with nutrient thresholds and 13 different taglines that can be used in food labeling •Products with this logo indicate they are lower in total fat, saturated fat, sodium, and sugar and higher in dietary fiber and calcium •For example, if a ready-to-eat breakfast meal contains ≤ 3 g saturated fat, ≤ 0.1 g trans fat, and ≤ 400 mg sodium per 100 g it would be labeled as “lower in saturated fat,” “lower in sodium” and “trans fat free”
Healthy Living Guarantee Mark 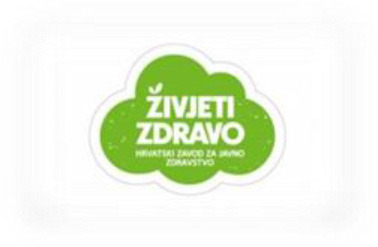	Croatia (2015)	•The Croatian Institute of Public Health •This logo is used for nine food groups: dairy products, fats and oils, vegetables, cereals, beverages, confectionery products, meat, fish, and fruits •The thresholds of nutrients are based on the daily recommended intake (RI) of selected nutrients for adults in the EU Directive •According to the threshold criteria of food groups, a convenient processed food should have a maximum of 3 g sugar, 1 g salt, and 2.5 g saturated fat per 100 g of food in the finished product
Healthier Choice 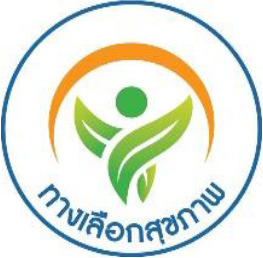	Thailand (2016)	•Thai Food and Drug Administration (Notification of MOPH (NO.373) B.E.2559 (2016) Re: Displaying Nutrition Symbols on Food Labels). •The Healthier Choices Logo helps customers identify healthier food choices effectively, including nine food categories •The foods are scored on a 0–5 scale depending on the contents of eight nutrients. To get the logo, a food item must have a minimum score of 20 out of 40
Healthier Choice Logo 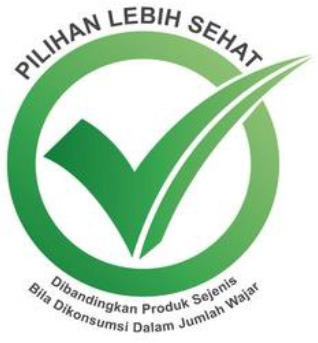	Indonesia (2019)	•Indonesian Food and Drug Administration (FDA) •Currently being applied to beverages and instant noodles/pasta •In 2021 the regulation was updated and now includes 20 different food groups including bakery goods, ready-to-eat snacks, etc. •This logo aligns with the Choices program and indicates the products have lower sugar, salt, and/or fat levels •To be eligible for the choice logo a 100 g ready-to-eat snack item must have a maximum of 400 mg of sodium and 20 g of total fat
Healthier Choice Logo Malaysia 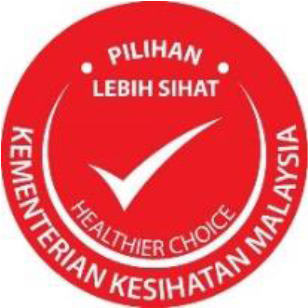	Malaysia (2017)	•Ministry of Health •The logo indicates that the product is healthier than other types of foods and is endorsed by the Ministry of Health Malaysia •It is applied to 41 product categories under 11 food groups and has specific nutrient threshold according to the product type and the food category
Healthier Choice Symbol 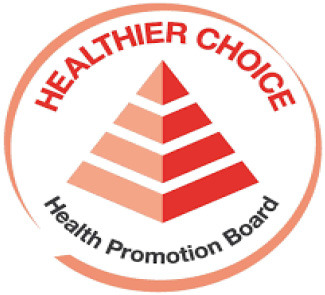	Singapore (2001)	•Ministry of Health •The Products with the symbol indicate they are generally lower in saturated fat, sodium, and sugar and higher in dietary fiber, calcium, and whole grains compared to similar products within the same food category •The thresholds for getting the tagline of “low in” the critical nutrients are ≤ 5 g of sugar, ≤ 3 g fat, ≤ 1.5 g saturated fat, ≤ 0.5 g trans fat, and ≤ 120 mg of salt per 100 g of food
Sangkap Pinoy Seal 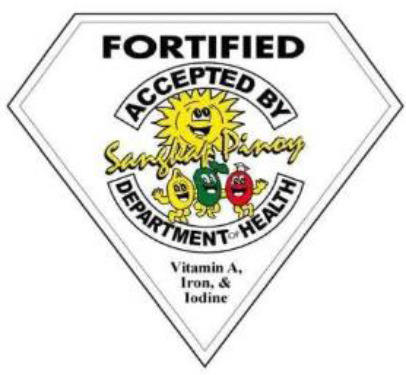	Philippines (2019)	•Department of Health •This logo works as a guide to help consumers to select fortified foods •The signs have three colors where yellow is for food products fortified with Vitamin A, Green is for food products fortified with iron, and Red is for food products fortified with iodine •No thresholds for the critical nutrients were found
**Hybrid mandatory FOPLs**		
Multiple Traffic Light Labeling 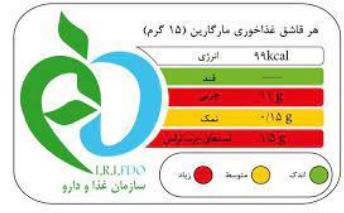	Iran (2015)	•Iranian Food and Drug Administration under Food and Beverage Labeling Regulation •The thresholds for critical nutrients for this scheme are ≤ 0.3 g of salt is regarded as a low level, >0.3 to ≤ 1.5 g is a medium level, and >1.5 g is considered a high level in 100 g •Similarly, in 100 g of food ≤ 5 g, >5 to ≤ 22.5 g, and >22.5 g of sugar are evaluated as low, medium, and high levels, respectively. •For trans-fat the levels per 100 g of food are low: ≤ 0.5 medium: >0.5 to ≤ 2, and high: >2 g
Traffic Light Coding System 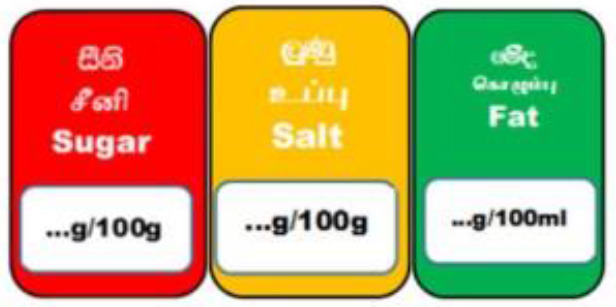	Sri Lanka (2019)	•Food Control Administration Unit, Ministry of Health (Only to beverage) •It is color-coded for sugar, salt, and fat •It is color-coded as green, amber, and red as low, moderate, and high •Over 22.5 g of sugar, 17.5 g of fat, 5 g of saturated fat, and 1.5 g of salt per 100 g is colored red. For green 100 g of food should contain ≤ 5 g of sugar, ≤ 3 g of fat, ≤ 1.5 g of saturated fat, and ≤ 0.3 g of salt •If the nutrients are in between red and green they should be marked as amber
**Hybrid voluntary FOPLs**		
Health star rating 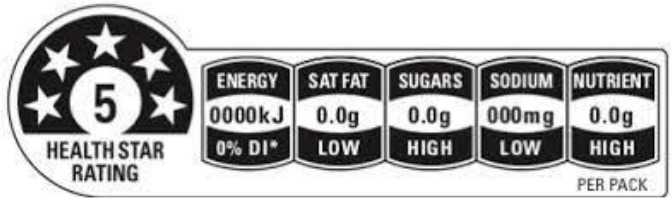	Australia (2014), New Zealand (2014)	•Initiatives jointly by the Australian and New Zealand government •The score is computed based on the content of positive nutrients (protein, dietary fiber, calcium, and certain vitamins and minerals) and risk nutrients such as salt, sugars, and fat •This system is based on an algorithm that assigns star ratings for foods ranging from $\frac{1}{2}$ star (least healthy) to 5 stars (most healthy) •No thresholds for the critical nutrients were found
Multiple Traffic Light Label 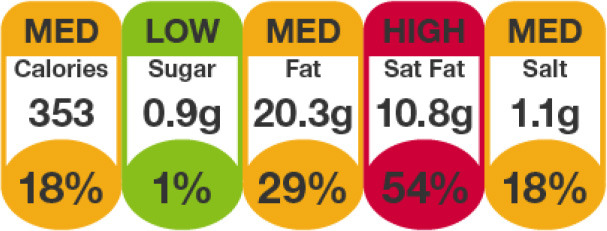	United Kingdom (2013), Ireland (2013)	•Food Standards Agency •This labeling scheme combines color coding and percentage reference intakes (RI) by UK standards and Article 35 of European Union (EU) Regulation 1169/2011 •If a food is high (red), medium (amber), or low (green) in nutrients of concern such as fat, saturated fat, sugar, and salt •The green and amber thresholds are set by the EU Reg 192/2006 whereas the red threshold is ≥25% of the RI value
Multiple Traffic Light 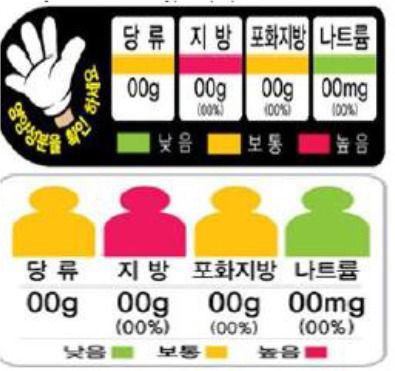	South Korea (2011)	•Ministry of Health under Special Act on Safety Control of Children's Dietary Life •-This labeling is used for children's food only •If a single serving size of food contains <3 g sugar, fat, <1.5 g saturated fat, and <120 mg salt it should be marked green. If >17 g sugar, >9 g fat, >4 g saturates, and >300 mg salt, it should be red. Amber should be between red and green
Multiple Traffic Light System 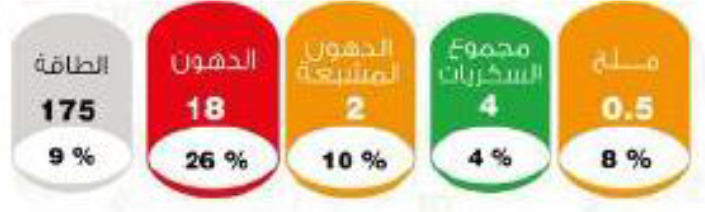	Saudi Arabia (2018)	•Saudi Food & Drug Authority (SFDA) •If 100 g of solid food has ≤ 0.3 g salt, ≤ 1.5 g saturated fat, and ≤ 5 g sugar, the color should be green. Similarly, red should be used for foods having >17.5 g fat, >5 g saturated fat, >22.5 g total carbohydrates, and >1.5 g salt •For 100 ml liquid, ≤ 1.5 g fat, ≤ 0.75 g saturated fat, ≤ 2.5 g sugar, and ≤ 0.3 g salt, should be coded green. In red, >8.75 g fat, 2.5 g saturated fat, >11.25 g sugar, and >0.75 g salt is the limit
• Traffic Light System • No Logo has been decided yet	United Arab Emirates (2019)	•National Program for Happiness and Wellbeing •All pre-packaged foods should be labeled and color-coded as green, amber, or red based on the fat, saturated fats, sugars, and salt content •No thresholds for critical nutrients were found
Multiple Traffic Light System 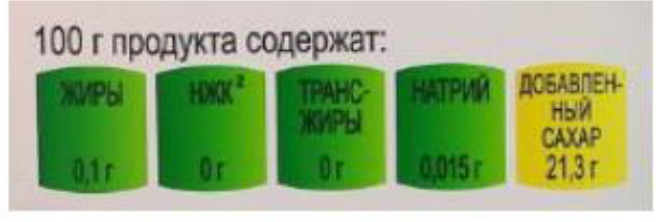	Russia (2018)	•Federal Research Center for Nutrition, Biotechnology, and Food Safety •This color-coded logo is applied to fish, meat, dairy, bread, canned fruits and vegetables, juices, and bread •The products are coded into red, yellow, and green based on salt, sugar, calories, and fat concentrations •No thresholds for critical nutrients were found
**Non-interpretative mandatory FOPLs**		
Guideline Daily Amount label (GDA) 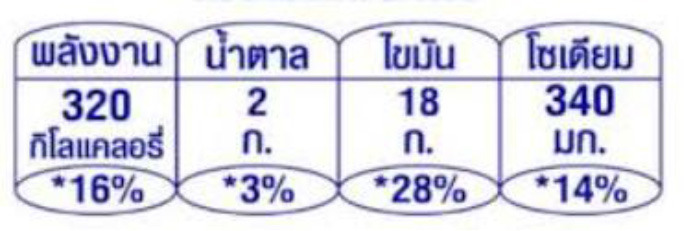	Thailand (2011)	•The Thai Food and Drug Administration (TFDA) introduced the GDA system in addition to the Nutrition Information Panel (NIP) •The label is mandatory for snack products and certain ready-to-eat foods •No thresholds for critical nutrients is set for this logo

Of the interpretive approaches, a single healthy food endorsement logo was adopted in 14 countries (Keyhole Logo in six countries and Healthier Choice Logo in eight countries), the Warning Label in 10 countries, the Nutri-score/Nutri-grade scheme in nine countries, and Traffic Light Labeling (TLL) in two countries. Of the hybrid approaches, the Multiple Traffic Light system was adopted in eight countries, and Health Star Rating in three countries. The single healthy food endorsement logo is broadly of two types—the Nordic Keyhole Logo, first introduced by Sweden, and the Healthier Choice Logo, initiated by the Choices Programme. The Healthier Choice Logos varied from country to country in their design and criteria for defining the healthiness of a food.

The warning label was the most common type of mandatory interpretive system, adopted mostly in the countries of the American region. Only Israel has the red warning label, and the rest of the country has black, although the shape of the warning label varies from country to country. Warning labels alert consumers to specific nutrients of concern, promoting informed decision-making.

The Nutri-score/Nutri-grade systems adopting countries were mostly in the European region, and the adoption was voluntary. The Nutri-score/Nutri-grade and TLL systems offer interpretive guidance to consumers about the nutritional quality of products through different color codes. Non-interpretative mandatory FOPLs, like Thailand's Guideline Daily Amounts (GDA), provide consumers with essential nutritional information without interpretative guidance.

Hybrid FOPL systems combine interpretative and non-interpretative elements. It incorporated the nutrient-specific information of GDA with an interpretive indicator of TLL in the Multiple Traffic Light system, and with the summary indicator in the Health Star Rating. South Korea first introduced the Multiple Traffic Light system, and Australia and New Zealand started the Health Star Rating system.

## 4 Discussion

In this document review, we compiled government-endorsed policies on nutrition labeling for packaged products available worldwide. Our review presents a comprehensive view of the nutrition labeling policies that may contribute to a reduction in population intake of unhealthy nutrients of concern. Following the Codex Alimentarius guideline (25), we reviewed policies on nutrient and ingredient declaration, nutrition claims, and supplementary nutrition information which focused on front-of-pack labeling (FOPL). Mandatory nutrient declaration on food packages is a key prerequisite for establishing FOPL and other nutrition policies (26–28). This review found that the declaration of ingredients and nutrients of packaged food products on their pack label is mandatory in 95 countries, 71 countries have rules for making a nutrient claims about the product, and 44 countries have FOPL policies. Among the countries having FOPL policies, 16 countries have adopted it as mandatory and 30 as voluntary. Two countries, Thailand and Singapore, have both voluntary and mandatory FOPL policies. Thailand has a mandatory Guideline Daily Amount (GDA) system for certain snack foods high in sugar, fat, and sodium, while a voluntary Healthier Choice Logo is used for categories such as snacks, beverages, dairy products, and ready-to-eat meals. Similarly, Singapore mandates the Nutri-Grade label for pre-packaged beverages with higher sugar and fat content, while the Healthier Choice Symbol remains voluntary for a wide range of food categories, including beverages, snacks, cooking oils, and sauces. Of the countries with mandatory FOPL policy, Bolivia, Canada, and Venezuela have adopted it as mandatory but have not implemented it until our review period. Venezuela is already in the implementation process and it will be implemented by December 2024 (29) and the food industry of Canada has until 1 January 2026 to implement it (30). Bolivia is contemplating transitioning from its interpretive-only Nutritional Traffic Light system to a warning label scheme to enhance consumer awareness of nutritional content.

The necessity of nutrition labeling was raised with the increasing availability of processed food products from the mid-20th century onward to ensure food quality and inform consumers. In 1972, the US FDA first proposed a regulation to declare nutritional information on the pack label. Later, Codex Alimentarius formulated guidelines on nutrition labeling in 1985 (31). The Codex guideline is mainly accepted and adopted by countries worldwide. In all countries with nutrient declaration policies, the list of ingredients must be included on the pack label, together with the declaration of nutrient components and their amounts. Different ministries and authorities —like the Ministry of Health, Ministry of Food, Ministry of Industry, and Municipalities— implemented this policy of nutrient declaration in different countries worldwide. The majority of the countries that have a policy on nutrition claims have adopted it in the same policy of nutrient declaration. Regarding nutrition claims, some countries have mentioned that any claim should be based on scientific evidence and the actual amount of content, whereas in many countries, specifications for different claims—such as free, low, high, enrich, etc.—have been documented in the regulation. Setting the specification for nutrition claims included many nutrients beyond the nutrients of concern. We excluded health claim-related policies from this review as health claims have a diverse dimension in practice, are confusing for consumers most of the time, and are difficult to formulate in regulations.

In 2004 WHO proposed nutritional labeling in accordance with Codex Alimentarius guidelines as part of its Global Strategy on Diet, Physical Activity and Health (32). The nutrient declaration aims in informing consumers about the specific nutrient content of foods, although it can be difficult to understand (26). In addition to being difficult to understand, barriers to using nutrition labels include illiteracy, lack of awareness, and distrust (28). A significant milestone in nutrition labeling was achieved during the WHO technical meeting on FOPL in December 2015, where a set of guiding principles for FOPL was established. These principles were further developed and updated in June 2017 (26). FOPL creates a positive food environment by supporting consumers in making healthier food choices quickly in a complex food environment and promoting food reformulation (27, 31, 33). The FOPL policy has been adopted in most countries as a voluntary approach; Ecuador was the first country that made it mandatory in 2014. Because of regulatory limitations, none of the EU countries could formulate any mandatory FOPL policies (24, 34).

Interpretive models of FOPL have been adopted more in recent years among the available schemes since evidence suggests that they are more effective in promoting healthy dietary habits. WHO Guiding Principles and Framework Manual (26) recommended interpretative labels for FOPL, especially those that use interpretational visual aids to minimize numerical information, as the best approach to aid consumers' comprehension of FOPL. They require less health and nutrition literacy, require less time, and help consumers in making quick decisions regarding the healthiness of the product. Among the interpretative FOPLs, warning labels have been adopted in 10 countries as mandatory FOPL policies. Finland first introduced a warning label to warn consumers against the high sodium content of a food; Chile was the first country in adopting a mandatory Warning Label to make consumers aware of the nutrients of concern and protect them from the harm of those nutrients (34).

Research has shown that mandatory FOPLs are more effective than voluntary systems in improving public health outcomes by providing consistent and clear nutritional information (35). A mandatory approach prevents food companies from selectively disclosing only favorable information, thereby reducing misleading marketing practices (36). Also, to avoid negative labeling manufacturers reformulate their products just below the nutrient cutoff, with common reformulated nutrients being sugar and sodium (37). Evaluations of Chile's mandatory warning labeling policy have shown significant declines in high-in nutrients of concern (sugar, salt, trans fat) in packaged food and beverage purchases, including a 20.2% relative reduction in sugar and a 13.8% relative reduction in sodium (38). Additional results show significant reformulation by the industry to avoid the warning label (39). However, when FOPL compliance is left voluntary, these labels often appear on some products but are absent from others. Furthermore, there is evidence that food companies selectively apply labels to healthier products while omitting them from less healthy options (36, 37). The review reveals significant variation in FOPL policies across countries, especially regarding nutrient thresholds. These differences are influenced by diverse dietary patterns, health priorities, and regulatory contexts. For example, countries with high rates of diet-related diseases may set stricter thresholds, while others adopt more moderate ones to fit local habits and industry practices. Gradual implementation of these thresholds allows manufacturers time to reformulate products and helps consumers adapt to new labeling practices. Understanding these variations is key to develop more effective and globally harmonized FOPL strategies.

The attempt to label healthier foods with an easy-to-interpret symbol is far older; in 1989, Sweden endorsed the “Keyhole” logo for the first time (31). Although the healthy food endorsement logo was the first FOPL system adopted, it is easy to manipulate and less effective in protecting consumers from harmful nutrients. Evaluating the effectiveness and comparing different types of FOPL was beyond the scope of our review, and there is ample evidence for such comparison. During our review, we explored that there are some industry-initiated initiatives for healthy food endorsement FOPL scheme. An industry-supported voluntary organization is working with various non-government and government organizations and agencies to promote such schemes, especially in the African and Asian continents. Adoption of voluntary FOPL in Zambia, Indonesia, Malaysia, Philippines is the outcome of such efforts, which we included in this review because of the government endorsement. We excluded similar policies of more than ten countries, which are promoted by different non-government organizations of respective countries (27, 39–42). Adoption of those interpretative FOPL schemes can create a “health halo” making packaged foods appear healthy by including some positive nutrients despite containing some high levels of harmful nutrients of concern (43).

Strengths of this document review include our attempt to compile all existing government-endorsed nutrition labeling policies, especially he elaborated documentation of the policies on FOPL including the adopted schemes throughout the world. It included the declaration of key nutrient and ingredient lists, which is crucial for establishing further nutrition policies for NCD risk reduction and health promotion, like front-of-package labeling (FOPL) adoption, and setting maximum targets for nutrients of concern in packaged products. The limitations of this article are the absence of an in-depth analysis of each policy, a description of the step-by-step progress toward the current policy environment, and the exclusion of studies on the effectiveness of the various policies and programs. Moreover, in this review, we looked into two databases only, and there might be other uncovered similar policies in other databases. In addition, we did not identify the specific threshold levels for FOPLs in all of the reviewed policies, and no quality assessment scale was employed during this review.

## 5 Conclusion

This document review aims to provide documentation of the available government-endorsed nutrition labeling schemes adopted by countries worldwide. Nutrition labeling has been regarded as one of the most important policy solutions for tackling the burden of obesity and diet-related NCDs. To prevent death and disabilities from NCDs, countries around the world have been adopting mandatory nutrition labeling schemes to limit the purchase of foods high in unhealthy nutrients of concern. As of late 2023, 95 countries have adopted mandatory declaration of ingredients and nutrients, and 71 countries have adopted policies to regulate nutrient claims. For an easier understanding of nutrition information on food packaging, FOPL has been adopted by 44 countries, including interpretative, non-interpretative, and hybrid schemes, of which, 16 countries have adopted mandatory FOPL schemes. Over the last few years, there has been a strong global momentum for developing mandatory nutrition labeling policies, which should be continued using available experiences and evidence on the best standard of practice. Local evidence from scientifically sound robust studies may be required in selecting and designing the most effective, well-understandable, and appropriate FOPL schemes in a country-specific context. A highly cautionary approach should be followed during policy adoptions to avoid the nuance of industry-initiated misguidance and their interference.
